# (2R*,6S*)-*tert*-Butyl 2,6-bis­(hydroxy­meth­yl)morpholine-4-carboxyl­ate

**DOI:** 10.1107/S1600536810017599

**Published:** 2010-06-16

**Authors:** Qian Chen, Bojun Li, Guangxin Xia

**Affiliations:** a Shanghai Institute of Materia Medica, Chinese Academy of Sciences, Shanghai 201203, People’s Republic of China; b Central Research Institute, Shanghai Pharmaceutical Group Co. Ltd, 555 Zuchongzhi Road, Shanghai 201203, People’s Republic of China

## Abstract

In the title compound, C_11_H_21_NO_5_, the H atoms of the hydr­oxy groups are disordered over two positions, each in a 1:1 ratio. In the crystal, inter­molecular O—H⋯O hydrogen bonds link pairs of mol­ecules into centrosymmetric dimers. Weak inter­molecular O—H⋯O inter­actions further link these dimers into chains extended in the [100] direction.

## Related literature

For details of the synthesis of 2,6-disubstituted morpholines, see: Dave & Sasaki (2004 ▶); Lupi *et al.* (2004 ▶).
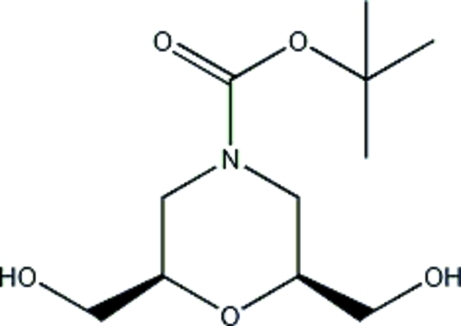


## Experimental

### 

#### Crystal data

C_11_H_21_NO_5_
*M* *_r_* = 247.29
Monoclinic, 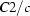

*a* = 21.909 (3) Å
*b* = 5.6643 (8) Å
*c* = 22.510 (3) Å
β = 107.612 (3)°
*V* = 2662.5 (7) Å^3^
*Z* = 8
Mo *K*α radiation
μ = 0.10 mm^−1^
*T* = 293 K
0.45 × 0.34 × 0.21 mm

#### Data collection

Bruker SMART CCD area-detector diffractometer
6675 measured reflections
2476 independent reflections
1699 reflections with *I* > 2σ(*I*)
*R*_int_ = 0.086

#### Refinement

*R*[*F* ^2^ > 2σ(*F* ^2^)] = 0.066
*wR*(*F* ^2^) = 0.199
*S* = 1.06
2476 reflections
173 parameters
4 restraints
H atoms treated by a mixture of independent and constrained refinement
Δρ_max_ = 0.53 e Å^−3^
Δρ_min_ = −0.38 e Å^−3^

### 

Data collection: *SMART* (Bruker, 2002 ▶); cell refinement: *SAINT* (Bruker, 2002 ▶); data reduction: *SAINT*; program(s) used to solve structure: *SHELXS97* (Sheldrick, 2008 ▶); program(s) used to refine structure: *SHELXL97* (Sheldrick, 2008 ▶); molecular graphics: *SHELXTL* (Sheldrick, 2008 ▶); software used to prepare material for publication: *SHELXTL*.

## Supplementary Material

Crystal structure: contains datablocks I, global. DOI: 10.1107/S1600536810017599/cv2712sup1.cif

Structure factors: contains datablocks I. DOI: 10.1107/S1600536810017599/cv2712Isup2.hkl

Additional supplementary materials:  crystallographic information; 3D view; checkCIF report

## Figures and Tables

**Table 1:** Hydrogen-bond geometry (Å, °)

*D*—H⋯*A*	*D*—H	H⋯*A*	*D*⋯*A*	*D*—H⋯*A*
O3—H3*D*⋯O3^i^	0.82 (3)	2.02 (3)	2.809 (4)	164 (6)
O2—H2*E*⋯O3^i^	0.86 (3)	1.99 (3)	2.849 (3)	176 (5)
O2—H2*D*⋯O4^ii^	0.82 (4)	2.48 (5)	3.269 (4)	163 (6)

Symmetry codes: (i) 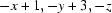; (ii) 
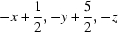
.

**Fig. 1.**
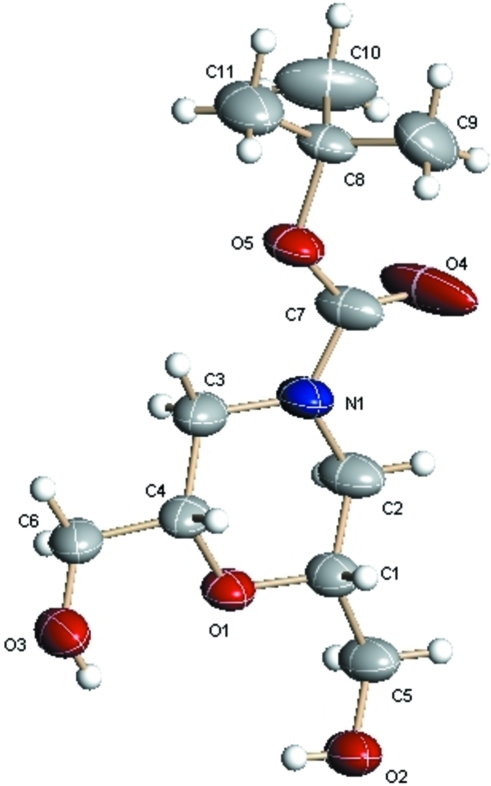
The molecular structure of (I) showing the atomic numbering and 30% probability displacement ellipsoids. For each of two disordered H atoms (bound to O2 and O3) only one position is shown.

## supplementary crystallographic information

### Comment

Morpholine and its derivatives have been widely investigated (Lupi *et al.*, 2004; Dave &Sasaki, 2004) due to their importance in the search of new therapeutically and biologically active compounds. In the present paper, we report the crystal structure of the title compound, (I).

In (I) (Fig. 1), the H atoms of two hydroxy groups are disordered over two positions each in a ratio 1:1. In the crystal, intermolecular O—H···O hydrogen bonds (Table 1) link two molecules into centrosymmetric dimer. Weak intermolecular O—H···O interactions [O···O 3.269 (4) Å] (Table 1) link further these dimers into chains extended in direction [100].

### Experimental

A mixture of (*S*)-(+)-benzyl glycidyl ether(9.84 g,60 mmol) and benzylamine(3.21 g,30 mmol) was heated with stirring at 60 centidegrees for 16 h. After being cooled to room temperature, an oil crude product (A) was obtained. Under ice-bath cooling, compound A (4.36 g,10 mmol) was dissolved in dry tetrahydrofuran (90 mL), 60% NaOH (1.0 g,25 mmol) was added over 15 minutes. The reaction mixture was stirred at 0 centidegrees for 30 minutes.Then a solution of TsCl(1.9 g,10 mmol) in dry THF(10 mL) was added dropwise over 30 minutes. After 10 min the solution was allowed to react at rt for 30 minutes and then heated at 50 centidegree until complete (usually about 2 h). After addition of an appropriate volume of 100 mL water, the aqueous layer was extracted three times with ethyl ether (30 mL). The combined organic layers were dried over anhydrous sodium sulfate and the solvent was removed and gave a yellow oil product(II). A solution of product II(2.09,5 mm mol) and acetic acid(10 mL) in methanol (30 mL) was treated with 10%Pd/C(200 mg) and then hydrogenated until complete(24 h). The catalyst was filtered and the solvent removed under reduced pressure.The pure product(III) was obtained. Product III was reacted with Boc anhydride to gave the target product. The target product was recrystallized from dry ether. Colourless crystals suitable for single crystal X-ray diffraction were obtained.

### Refinement

H atoms bonded to O2 and O3 were each positioned in two possible idealized positions with occupancies fixed to 0.5, and were isotropically refined with the O—H bond length restrained to 0.82 (3) Å. C-bound H atoms were geometrically positioned (C—H = 0.96-0.98 Å)  and treated as riding, with U_iso_(H)=1.2-1.5 U_eq_(C).

### Crystal data

**Table:**

C_11_H_21_NO_5_	*F*(000) = 1072
*M**_r_* = 247.29	*D*_x_ = 1.234 Mg m^−^^3^
Monoclinic, *C*2/*c*	Mo *K*α radiation, λ = 0.71073 Å
*a* = 21.909 (3) Å	Cell parameters from 1734 reflections
*b* = 5.6643 (8) Å	θ = 4.6–44.5°
*c* = 22.510 (3) Å	µ = 0.10 mm^−^^1^
β = 107.612 (3)°	*T* = 293 K
*V* = 2662.5 (7) Å^3^	Prismatic, colourless
*Z* = 8	0.45 × 0.34 × 0.21 mm

### Data collection

**Table:**

Bruker SMART CCD area-detector diffractometer	1699 reflections with *I* > 2σ(*I*)
Radiation source: fine-focus sealed tube	*R*_int_ = 0.086
graphite	θ_max_ = 25.5°, θ_min_ = 1.9°
φ and ω scans	*h* = −26→25
6675 measured reflections	*k* = −6→6
2476 independent reflections	*l* = −27→19

### Refinement

**Table:**

Refinement on *F*^2^	Primary atom site location: structure-invariant direct methods
Least-squares matrix: full	Secondary atom site location: difference Fourier map
*R*[*F*^2^ > 2σ(*F*^2^)] = 0.066	Hydrogen site location: inferred from neighbouring sites
*wR*(*F*^2^) = 0.199	H atoms treated by a mixture of independent and constrained refinement
*S* = 1.06	*w* = 1/[σ^2^(*F*_o_^2^) + (0.0978*P*)^2^ + 0.9544*P*] where *P* = (*F*_o_^2^ + 2*F*_c_^2^)/3
2476 reflections	(Δ/σ)_max_ = 0.028
173 parameters	Δρ_max_ = 0.53 e Å^−^^3^
4 restraints	Δρ_min_ = −0.38 e Å^−^^3^

### Special details

**Table:**

Geometry. All esds (except the esd in the dihedral angle between two l.s. planes) are estimated using the full covariance matrix. The cell esds are taken into account individually in the estimation of esds in distances, angles and torsion angles; correlations between esds in cell parameters are only used when they are defined by crystal symmetry. An approximate (isotropic) treatment of cell esds is used for estimating esds involving l.s. planes.
Refinement. Geometry. All esds (except the esd in the dihedral angle between two l.s. planes) are estimated using the full covariance matrix. The cell esds are taken into account individually in the estimation of esds in distances, angles and torsion angles; correlations between esds in cell parameters are only used when they are defined by crystal symmetry. An approximate (isotropic) treatment of cell esds is used for estimating esds involving l.s. planes. Refinement. Refinement of F2 against ALL reflections. The weighted *R*-factor wR and goodness of fit *S* are based on F2, conventional *R*-factors *R* are based on *F*, with *F* set to zero for negative F2. The threshold expression of F2 > σ(F2) is used only for calculating *R*-factors(gt) etc. and is not relevant to the choice of reflections for refinement. *R*-factors based on F2 are statistically about twice as large as those based on *F*, and *R*- factors based on ALL data will be even larger.

### Fractional atomic coordinates and isotropic or equivalent isotropic displacement parameters (Å^2^)

**Table:**

	*x*	*y*	*z*	*U*_iso_*/*U*_eq_	Occ. (<1)
O1	0.41734 (8)	1.2127 (3)	0.00900 (7)	0.0487 (5)	
O2	0.33480 (10)	1.4453 (4)	−0.09361 (9)	0.0668 (6)	
O3	0.53146 (9)	1.4619 (4)	0.06357 (9)	0.0638 (6)	
O4	0.28864 (15)	0.7185 (5)	0.10144 (10)	0.1312 (14)	
O5	0.38194 (9)	0.7747 (3)	0.17686 (8)	0.0598 (6)	
N1	0.36921 (10)	0.9179 (4)	0.08274 (10)	0.0584 (6)	
C1	0.34976 (12)	1.1868 (5)	−0.00456 (11)	0.0503 (7)	
H1	0.3335	1.3098	0.0172	0.060*	
C2	0.33469 (15)	0.9479 (5)	0.01673 (12)	0.0650 (8)	
H2A	0.2890	0.9344	0.0102	0.078*	
H2B	0.3472	0.8254	−0.0074	0.078*	
C3	0.43769 (13)	0.9642 (5)	0.09948 (13)	0.0622 (8)	
H3A	0.4584	0.8401	0.0829	0.075*	
H3B	0.4558	0.9643	0.1445	0.075*	
C4	0.44998 (11)	1.1985 (4)	0.07397 (10)	0.0477 (6)	
H4	0.4348	1.3251	0.0956	0.057*	
C5	0.32122 (14)	1.2194 (5)	−0.07387 (12)	0.0623 (8)	
H5A	0.3381	1.0993	−0.0953	0.075*	
H5B	0.2752	1.1985	−0.0851	0.075*	
C6	0.51984 (12)	1.2342 (5)	0.08203 (12)	0.0566 (7)	
H6A	0.5442	1.2103	0.1254	0.068*	
H6B	0.5341	1.1185	0.0573	0.068*	
C7	0.34176 (15)	0.7944 (5)	0.11901 (12)	0.0621 (8)	
C8	0.36300 (13)	0.6503 (5)	0.22584 (11)	0.0527 (7)	
C9	0.30285 (16)	0.7518 (6)	0.23374 (18)	0.0880 (11)	
H9A	0.3061	0.9208	0.2358	0.132*	
H9B	0.2968	0.6926	0.2715	0.132*	
H9C	0.2670	0.7071	0.1989	0.132*	
C10	0.3569 (2)	0.3929 (6)	0.21181 (18)	0.1117 (15)	
H10A	0.3211	0.3662	0.1753	0.168*	
H10B	0.3503	0.3098	0.2465	0.168*	
H10C	0.3953	0.3367	0.2045	0.168*	
C11	0.41842 (16)	0.7042 (7)	0.28251 (14)	0.0860 (11)	
H11A	0.4570	0.6392	0.2773	0.129*	
H11B	0.4108	0.6357	0.3186	0.129*	
H11C	0.4230	0.8721	0.2878	0.129*	
H2E	0.3751 (10)	1.474 (9)	−0.083 (2)	0.050 (15)*	0.50
H3E	0.5706 (11)	1.495 (9)	0.074 (3)	0.056 (16)*	0.50
H2D	0.303 (2)	1.529 (11)	−0.104 (3)	0.09 (3)*	0.50
H3D	0.507 (2)	1.477 (10)	0.0285 (13)	0.055 (16)*	0.50

### Atomic displacement parameters (Å^2^)

**Table:**

	*U*^11^	*U*^22^	*U*^33^	*U*^12^	*U*^13^	*U*^23^
O1	0.0563 (11)	0.0596 (11)	0.0309 (9)	−0.0055 (8)	0.0143 (8)	0.0060 (7)
O2	0.0609 (14)	0.0802 (15)	0.0548 (12)	−0.0088 (12)	0.0109 (11)	0.0241 (11)
O3	0.0524 (12)	0.0870 (15)	0.0460 (12)	−0.0197 (10)	0.0059 (10)	0.0093 (10)
O4	0.147 (2)	0.169 (3)	0.0511 (13)	−0.123 (2)	−0.0095 (14)	0.0263 (15)
O5	0.0710 (12)	0.0721 (12)	0.0389 (10)	−0.0173 (9)	0.0205 (9)	0.0112 (8)
N1	0.0625 (14)	0.0725 (15)	0.0412 (12)	−0.0083 (11)	0.0172 (10)	0.0176 (11)
C1	0.0550 (15)	0.0575 (16)	0.0366 (13)	−0.0072 (12)	0.0113 (12)	0.0049 (11)
C2	0.083 (2)	0.0706 (19)	0.0403 (15)	−0.0186 (15)	0.0171 (14)	0.0078 (13)
C3	0.0615 (17)	0.0780 (19)	0.0519 (16)	0.0106 (14)	0.0243 (14)	0.0236 (14)
C4	0.0539 (15)	0.0601 (15)	0.0305 (12)	0.0048 (11)	0.0149 (11)	0.0068 (11)
C5	0.0687 (17)	0.0713 (18)	0.0415 (15)	−0.0159 (14)	0.0084 (13)	0.0121 (13)
C6	0.0551 (16)	0.0737 (19)	0.0406 (14)	0.0023 (13)	0.0143 (12)	0.0147 (13)
C7	0.086 (2)	0.0583 (16)	0.0398 (15)	−0.0304 (15)	0.0167 (14)	0.0014 (12)
C8	0.0714 (17)	0.0535 (15)	0.0382 (14)	−0.0117 (12)	0.0242 (13)	0.0085 (11)
C9	0.084 (2)	0.101 (3)	0.096 (3)	−0.0023 (18)	0.053 (2)	0.019 (2)
C10	0.212 (5)	0.0526 (19)	0.087 (3)	−0.011 (2)	0.070 (3)	0.0050 (19)
C11	0.087 (2)	0.119 (3)	0.0514 (18)	−0.0166 (19)	0.0197 (17)	0.0188 (18)

### Geometric parameters (Å, °)

**Table:**

O1—C4	1.422 (3)	C3—H3A	0.9700
O1—C1	1.426 (3)	C3—H3B	0.9700
O2—C5	1.415 (3)	C4—C6	1.499 (3)
O2—H2E	0.858 (19)	C4—H4	0.9800
O2—H2D	0.82 (2)	C5—H5A	0.9700
O3—C6	1.402 (3)	C5—H5B	0.9700
O3—H3E	0.84 (2)	C6—H6A	0.9700
O3—H3D	0.817 (19)	C6—H6B	0.9700
O4—C7	1.191 (4)	C8—C10	1.489 (4)
O5—C7	1.338 (3)	C8—C9	1.497 (4)
O5—C8	1.470 (3)	C8—C11	1.503 (4)
N1—C7	1.347 (3)	C9—H9A	0.9600
N1—C3	1.456 (3)	C9—H9B	0.9600
N1—C2	1.459 (3)	C9—H9C	0.9600
C1—C2	1.505 (4)	C10—H10A	0.9600
C1—C5	1.506 (3)	C10—H10B	0.9600
C1—H1	0.9800	C10—H10C	0.9600
C2—H2A	0.9700	C11—H11A	0.9600
C2—H2B	0.9700	C11—H11B	0.9600
C3—C4	1.502 (4)	C11—H11C	0.9600
			
C4—O1—C1	112.38 (18)	C1—C5—H5A	109.2
C5—O2—H2E	112 (3)	O2—C5—H5B	109.2
C5—O2—H2D	112 (5)	C1—C5—H5B	109.2
H2E—O2—H2D	134 (6)	H5A—C5—H5B	107.9
C6—O3—H3E	113 (4)	O3—C6—C4	111.0 (2)
C6—O3—H3D	105 (4)	O3—C6—H6A	109.4
H3E—O3—H3D	125 (6)	C4—C6—H6A	109.4
C7—O5—C8	121.21 (19)	O3—C6—H6B	109.4
C7—N1—C3	123.4 (2)	C4—C6—H6B	109.4
C7—N1—C2	119.3 (2)	H6A—C6—H6B	108.0
C3—N1—C2	114.8 (2)	O4—C7—O5	125.6 (2)
O1—C1—C2	109.8 (2)	O4—C7—N1	123.9 (3)
O1—C1—C5	106.7 (2)	O5—C7—N1	110.5 (2)
C2—C1—C5	112.2 (2)	O5—C8—C10	109.7 (2)
O1—C1—H1	109.4	O5—C8—C9	111.3 (2)
C2—C1—H1	109.4	C10—C8—C9	112.0 (3)
C5—C1—H1	109.4	O5—C8—C11	101.6 (2)
N1—C2—C1	109.5 (2)	C10—C8—C11	112.1 (3)
N1—C2—H2A	109.8	C9—C8—C11	109.6 (3)
C1—C2—H2A	109.8	C8—C9—H9A	109.5
N1—C2—H2B	109.8	C8—C9—H9B	109.5
C1—C2—H2B	109.8	H9A—C9—H9B	109.5
H2A—C2—H2B	108.2	C8—C9—H9C	109.5
N1—C3—C4	110.5 (2)	H9A—C9—H9C	109.5
N1—C3—H3A	109.5	H9B—C9—H9C	109.5
C4—C3—H3A	109.6	C8—C10—H10A	109.5
N1—C3—H3B	109.6	C8—C10—H10B	109.5
C4—C3—H3B	109.5	H10A—C10—H10B	109.5
H3A—C3—H3B	108.1	C8—C10—H10C	109.5
O1—C4—C6	107.10 (19)	H10A—C10—H10C	109.5
O1—C4—C3	110.5 (2)	H10B—C10—H10C	109.5
C6—C4—C3	111.5 (2)	C8—C11—H11A	109.5
O1—C4—H4	109.2	C8—C11—H11B	109.5
C6—C4—H4	109.2	H11A—C11—H11B	109.5
C3—C4—H4	109.2	C8—C11—H11C	109.5
O2—C5—C1	112.0 (2)	H11A—C11—H11C	109.5
O2—C5—H5A	109.2	H11B—C11—H11C	109.5
			
C4—O1—C1—C2	−61.4 (3)	C2—C1—C5—O2	179.3 (2)
C4—O1—C1—C5	176.8 (2)	O1—C4—C6—O3	64.6 (3)
C7—N1—C2—C1	145.5 (3)	C3—C4—C6—O3	−174.3 (2)
C3—N1—C2—C1	−52.0 (3)	C8—O5—C7—O4	0.4 (5)
O1—C1—C2—N1	55.4 (3)	C8—O5—C7—N1	179.1 (2)
C5—C1—C2—N1	173.9 (2)	C3—N1—C7—O4	−165.7 (3)
C7—N1—C3—C4	−147.9 (3)	C2—N1—C7—O4	−4.8 (5)
C2—N1—C3—C4	50.5 (3)	C3—N1—C7—O5	15.6 (4)
C1—O1—C4—C6	−178.62 (19)	C2—N1—C7—O5	176.5 (2)
C1—O1—C4—C3	59.7 (3)	C7—O5—C8—C10	68.9 (4)
N1—C3—C4—O1	−52.3 (3)	C7—O5—C8—C9	−55.7 (3)
N1—C3—C4—C6	−171.3 (2)	C7—O5—C8—C11	−172.3 (3)
O1—C1—C5—O2	−60.5 (3)		

### Hydrogen-bond geometry (Å, °)

**Table:**

*D*—H···*A*	*D*—H	H···*A*	*D*···*A*	*D*—H···*A*
O3—H3D···O3^i^	0.82 (3)	2.02 (3)	2.809 (4)	164 (6)
O2—H2E···O3^i^	0.86 (3)	1.99 (3)	2.849 (3)	176 (5)
O2—H2D···O4^ii^	0.82 (4)	2.48 (5)	3.269 (4)	163 (6)

Symmetry codes: (i) −*x*+1, −*y*+3, −*z*; (ii) −*x*+1/2, −*y*+5/2, −*z*.
